# Exploring the antibacterial potential of *Clidemia hirta* leaf extract against the pathogenicity of *Pseudomonas aeruginosa: in vitro* and *in silico* approaches

**DOI:** 10.3389/fphar.2025.1555542

**Published:** 2025-03-12

**Authors:** Vignesh Murugesan, Pargovan Palanivel, Gokul Ramesh, Dwarakesh Ganesh, Helan Soundra Rani Michael, Shivakumar Bandhumy Lingam, Rathish Kumar Sivaraman

**Affiliations:** ^1^ Department of Biotechnology, Sri Ramakrishna College of Arts & Science, Coimbatore, Tamil Nadu, India; ^2^ Department of Biotechnology, Manonmaniam Sundaranar University, Tirunelveli, India

**Keywords:** *Clidemia hirta*, *Pseudomonas aeruginosa*, antibacterial activity, pathogenicity, penicillium binding protein

## Abstract

**Background:**

Multidrug-resistant bacterial pathogen *P. aeruginosa* has emerged as a significant global health challenge, underscoring the urgent need to identify and develop alternative therapeutic agents including plant natural products. In this study, the extract from *Clidemia hirta* plant extract was analyzed for antibacterial properties against *Pseudomonas aeruginosa* and component composition.

**Material and Methods:**

The plant extract was obtained from leaves of *C. hirta* and its antibacterial activity against *P. aeruginosa* was determined in Kirby-Bauer disc diffusion assay. In this assay, the activity of the extract was tested at two different concentrations of 50 and 100 μg/mL. The minimum inhibitory concentration (MIC) of the extract against *P. aeruginosa* was used with its MIC values against Vero cells to determine the selectivity index. GC-MS determined the phytochemical composition of the plant extract. The property of different extract components to bind the target receptor Penicillin Binding Protein 2a (7KIS) was assessed *in silico* studies including docking and molecular dynamics (MD) analyses. In these analyses, the stability and interaction dynamics of the Penicillin Binding Protein 2a (7KIS) protein complexed with selected extract components.

**Results:**

The plant extracts had antibacterial activity against *P. aeruginosa*, with inhibition zones measuring 13 mm and 19 mm for 50 and 100 μg/mL concentrations, respectively. The MIC of the plant extract was determined to be 20 μg/mL, while its selectivity index was 4.54, indicating its antibiotic potential. One extract component, 2, 4-di-tert-butylphenol compound holds a binding affinity of −6.2 kcal/mol in molecular docking studies. MD simulations revealed stable binding interactions between the 7KIS protein and the tested ligands, characterized by reduced atomic fluctuations and energetically favorable binding profiles.

**Conclusion:**

This study showed that *C. hirta* extract has a robust antibacterial potential against *P. aeruginosa*. Furthermore, GC-MS profiling molecular docking, and dynamic simulation data showed that such antibacterial potential might be attributed to its one component, 2, 4-di-tert-butylphenol. Further, *in vivo* and *in vitro* studies are needed to show the applicability of bioactive compounds from *C. hirta* in combating resistant bacterial pathogens.

## Graphical Abstract

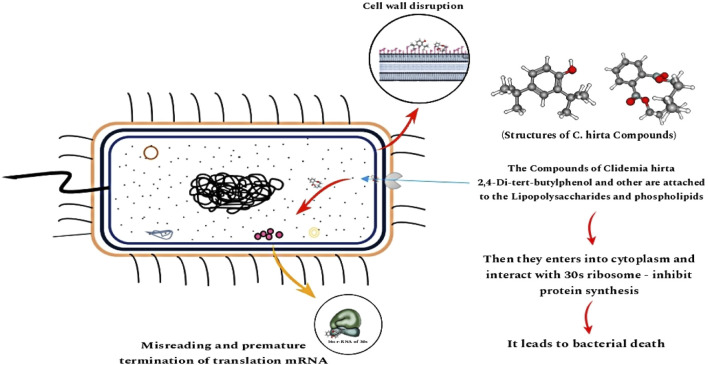

## 1 Introduction

*Pseudomonas aeruginosa* is a Gram-negative, rod-shaped bacterium classified within the Pseudomonadaceae family of the γ-proteobacteria class, responsible for infections, including urinary tract infections, chronic pulmonary infections, gastrointestinal disorders, and bloodstream infections. It presents a grave challenge in patients with cancer, cystic fibrosis, and burn injuries, often leading to a fatality rate of approximately 50%. Additional clinical concerns include skin and soft tissue infections, endocarditis, bacteremia, septicemia, external otitis, and ocular infections. *P. aeruginosa* typically involves various antibiotics, such as ceftazidime, ciprofloxacin, cefepime, carbapenems, and tobramycin. However, therapeutic efficacy frequently declines due to the development of antibiotic resistance, which arises from bacterial mutations that confer resistance to specific antimicrobial agents (Jones et al., 2022). Antibiotics such as cephalosporins, quinolones, macrolides, tetracyclines, and carbapenems are often ineffective due to these genetic adaptations. The broad-spectrum antibiotics known as aminoglycosides (Gentamicin, Amikacin, Tobramycin, Neomycin, and Streptomycin) are derived from actinomycetes and bacterial species and work against Gram-positive and Gram-negative bacteria. It proliferates efficiently in media containing acetate as a C source and (NH_4_)_2_SO_4_ as a N source. This organism is intrinsically resistant to high concentrations of salts, dyes, weak antiseptics, and a broad spectrum of commonly utilized antibiotics. *P. aeruginosa* has become an emergent opportunistic pathogen in clinical settings, rarely infecting intact, healthy tissues but capable of invading compromised tissues, particularly in individuals with immunodeficiencies. The bacterium synthesizes two soluble pigments: pyoverdine, a fluorescent pigment, and pyocyanin, a blue pigment. They work by attaching to phospholipids and lipopolysaccharides in the outer membranes of Gram-negative bacteria and teichoic acid and phospholipids in Gram-positive bacteria. This binding alters the membrane permeability, facilitating the entry of aminoglycosides into the cytoplasm, where they target the 30 S ribosomal subunit. The bacterium’s capacity to mis-translate proteins ultimately determines whether aminoglycosides will successfully bind to the ribosomes and inhibit protein synthesis (Webster and Shepherd, 2023; Krause et al., 2016). However, side effects such as kidney injury, hearing impairments, vestibular toxicity (Prayle et al., 2010), and depression of neuromuscular function may cause prolonged paralysis of respiratory muscles (Block and Blanchard, 2023). Researchers suggest green-based approaches using plant secondary metabolites with properties similar to aminoglycosides with zero side effects. In this study, such approaches may pave the path to a remarkable discovery against antibiotics for the pathogenic bacteria *P. aeruginosa* via *in vitro* and *in silico* methods.

*Miconia crenata*, the scientific name for *Clidemia hirta*, is a member of the Melastomataceae family. This shrub grows densely and usually reaches a height of 0.5–3 m, while it can grow up to 5 m in specific environments. The leaves are primarily greenish and have an oval form with pointed points (Judd et al., 2018). The local Malaysian tribe known as “senduduk bulu” used crushed leaves of *C. hirta* L. combined with saliva as a poultice on wounds to stop bleeding (Musa, 2007; Lopez et al., 2016) and to treat venom fever (Dianita et al., 2011; Kamarudin and Latiff, 2002), even though the plant is an invasive one that disturbs plantations and agricultural land. In Brazil, *Leishmania braziliensis* skin infections and several bacterial diseases are treated with *Clidemia* sp. (Franca et al., 1996; McClatchey, 1996). In early experiments to control bacterial typhus in Indonesia, where 37% of individuals experienced high fever symptoms in the 1990s, these ethnically based *C. hirta* plant extracts were employed as an antibacterial agent (Azad, 1990). Additionally, they frequently include strong antibacterial properties that help stop *Salmonella typhi* (Pratami et al., 2021). The ethanolic extract of *C. hirta* has demonstrated effective anti-proliferation against DLA cancer cell leaves (Narasimham et al., 2017; Dianita et al., 2011). *C. hirta* leaf extracts in 70% ethanol and aqueous form demonstrated antibacterial efficacy against *S. aureus* and *S. typhi*, respectively (Pratami et al., 2021). According to reports, *C. hirta* leaf extract possesses bacteriostatic properties against *Enterococci faecalis* and bactericidal properties against *P. aeruginosa* (Abrahim, 2010). According to Melendez and Capriles (2006), antibacterial substances can inhibit the growth or metabolism of bacteria (Pelezar and Chan, 1988) and increase their efficacy against *S. aureus*. Most of this research attests that this family inhibits bacterial growth (Gray, 1995). One of the primary antibacterial substances found in this plant is arjunolic acid, a triterpenoid saponin, according to El Abdellaoui et al. (2014). Additionally, its leaves have been shown to contain many phenolics and flavonoids (Murdiati et al., 1990), and its potential application as an antioxidant in cosmetics has been patented (Pauly and Moretti, 2003). Correspondingly, employing *in silico* studies such as molecular docking and simulation can augment the particular metabolites' strongest affinity towards the bacterial receptors and execute the antibacterial mechanisms against *P. aeruginosa*. Penicillin-binding protein 2a (PBP2a – target receptor) and plant-derived ligands were selected for docking analysis. Discovery Studio and AMDock were utilized, and the resulting affinity values indicated the strength of the interaction between the target protein and the ligands. A higher affinity suggests a stronger interaction, ultimately allowing for the assessment of whether the antimicrobial compounds in *C. hirta* could serve as effective protectants against bacterial infections.

## 2 Methods

### 2.1 Collection of plant sample

The leaves of *C. hirta* plant material were collected from Ooty Hills, Tamil Nadu, India. The leaves of the plant were surface sterilized, washed, shade-dried, and then powdered. Soxhlet apparatus was used for plant extraction. The extraction was prepared using 100 mL methanol with 20 g of plant sample (Okoduwa et al., 2016).

### 2.2 Preparation of plant extracts

The extraction process for *C. hirta* leaves was conducted using methanol as the solvent. 10 g of powdered leaf material were macerated in 300 mL of methanol for 3 days under ambient conditions. Following the extraction, the resulting filtrate was clarified by removing the residual particulate matter using Whatman filter paper, as Abubakar and Haque (2020) described.

### 2.3 GC-MS analysis

The GC-MS analysis was performed using a combined 7890A gas chromatograph system (Agilent 19,091-433 HP, USA) and mass spectrophotometer with an HP-5 MS. The fused silica column was interfaced with a 5675C Inert MSD with Triple-Axis detector (5% phenyl methyl siloxane, 30.0 m × 250 μm, film thickness 0.25 μm). Helium gas was used as the carrier gas, and its column velocity flow rate was set at 1.0 mL per minute. Additional specifications for GC-MS include an ion source temperature of 250°C, an interface temperature of 300°C, a pressure of 16.2 psi, a time of 1.8 mm, and a split mode of 1 μL injector at 300°C for injection temperature and a split ratio of 1:50. At a rate of 4°C per minute, the temperature in the column rose from 36°C for 5 min to 150°V. Over 5 min, the temperature was raised to 250°C at 20 °C/min 47.5 min were needed for the whole elution. Each component’s relative percent amount was calculated by comparing its average peak area to the element’s total areas (Olivia et al., 2021).

### 2.4 Antibacterial susceptibility test

The antibacterial activity against *P. aeruginosa* was assessed using the disc diffusion method. A sterilized cotton swab spreads the bacterial strains on nutrient agar plates. Two concentrations (50 and 100 μg/mL) of *C. hirta* leaf extracts were applied to the disk. DMSO served as the negative control, and Kanamycin was the positive control. For 24 h, the plates were incubated at 37°C. The zone of inhibition was measured to assess the antibacterial activity (Rahayu et al., 2022; Schadich et al., 2022). The procedure was repeated twice with the same concentration of leaf extracts.

### 2.5 Minimum inhibitory concentration

The *C. hirta* leaf extract was dissolved in a suitable solvent, and different concentrations were used, such as 20, 40, 60, 80, and 100 μL, respectively. To obtain a bacterial suspension with a density of 5 × 10^5^ CFU/mL for broth microdilution, 0.5 McFarland suspension should be diluted 100× to a density of 10^6^ CFU/mL (9.9 mL broth +0.1 mL 0.5 McFarland suspension) and then poured to wells containing the appropriate antibiotic concentrations in the broth (50 μL bacterial inoculum +50 liquid medium with antibiotic) (EUCAST, 2003). For validation, both positive and negative controls were used. Bacterial growth was evaluated visually using a spectrophotometer to measure absorbance at 630 nm, and the tubes were incubated for 24 h at 37°C. The minimum inhibitory concentration (MIC) was defined as the concentration at which observable bacterial growth was inhibited (Rahayu et al., 2022). The selectivity index (S.I.) was calculated regarding the CC_50_ obtained from the study of Aristizabal et al. (2013). SI > 10 → Highly selective (potentially safe for therapeutic use). SI between 3 and 10 → Moderately selective. SI < 3 → Low selectivity (may not be ideal for therapeutic use). SI was calculated using the formula.

$$Selectivity\;Index\;\left(SI\right)=\frac{LC50}{MIC}$$

### 2.6 Molecular docking

Molecular docking and simulation are performed using AMDock and GROMACS, among others. AMDock and Discovery Studio are used for molecular docking, and PubChem websites to download chemical compounds in various dimensions, such as 1D, 2D, and 3D (https://pubchem.ncbi.nlm.nih.gov/). Discovery Studio software removes the water molecules and changes the format of the proteins and compounds. Finally, AMDock is used for docking; here, the hetero method is used. 3D structure of the ligands and standard retrieved from PubChem were saved in PDB format (pdbqt) in rcsb.org to dock against the penicillin-binding protein 2a target protein named as 7KIS (Crystal structure of *P. aeruginosa* PBP2 in complex with WCK 5153 - enhancer of β-lactamase). The grid box was adjusted to cover the active sites of the target protein and the binding ligand (X = 12, Y = 12, and Z = 12) with spacing 1 Å. The binding affinity during the target protein and ligand interactions with a minimum free binding energy was determined using the software Autodock Vina 4.2 (offline open-source software). The confirmation and target protein interactions like hydrophilic interactions, H bonding, and van der Waals forces were analyzed using Discovery Studio Visualizer 2022 (Na’abba et al., 2022).

### 2.7 Simulation

A computational method called molecular dynamics (MD) simulation uses Newton’s equations of motion to examine how atoms move within molecules. In this instance, the popular and reliable program GROMACS was employed to carry out the MD simulation. Reducing the protein-ligand complex is the first step to consider in the simulation process. The steepest descent algorithm is used to iteratively modify the complex’s atomic coordinates to reduce the system’s potential energy. Following minimization, the SPC water model was used to solvate the complex in a periodic water box. The straightforward SPC water model represents single-point charge water molecules. It frequently serves as the foundation for more intricate water models. Additionally, by adding the proper concentration of sodium and chloride ions, the complex was kept at a salt concentration of 0.15 M. Before undergoing a 100 ns (nanoseconds) production run in the NPT ensemble, the resultant complex underwent an NPT (constant pressure, constant temperature) equilibration phase. Systems containing temperature and pressure frequently present in biological systems are simulated using the NPT ensemble. The GROMACS software package’s capabilities, which include the protein Root Mean Square Deviation (RMSD), Root Mean Square Fluctuation (RMSF), Radius of Gyration (RG), Solvent Accessible Surface Area (SASA), and H-Bond, were then used to analyze the simulation’s trajectory. Using these experiments, researchers can examine the general form, flexibility, and interactions with the surrounding solvent of the simulated system, among other structural and dynamic aspects. The target’s complex underwent MM-PBSA calculations for this experiment, and each complex’s GROMAC trajectory gained 50 ns as the final. GROMAC was initially utilized to generate topology files and incorporate an explicit solvent to prepare complex structures for computation. Following the configuration of the MM-PBSA computation in the g_ MMPBSA program, the energy decomposition was performed on each complex during the last 100 ns of the trajectory. The gathered energy components were analyzed to determine the binding affinity and the contributions of different energy terms to the total binding energy.

## 3 Result

### 3.1 Antibacterial susceptibility test

The inhibition zones, indicative of antibacterial efficacy, were measured and recorded as the diameter of clear zones formed around the discs. In this study, the *C. hirta* leaf extracts with 100 µL concentration showed sensitivity with 19 mm diameter. In contrast, the extract of 50 µL concentration exhibited 13 mm in diameter, nearer to the positive control of Kanamycin antibiotic (16 mm).

### 3.2 Minimum inhibitory concentration

**FIGURE 1:**
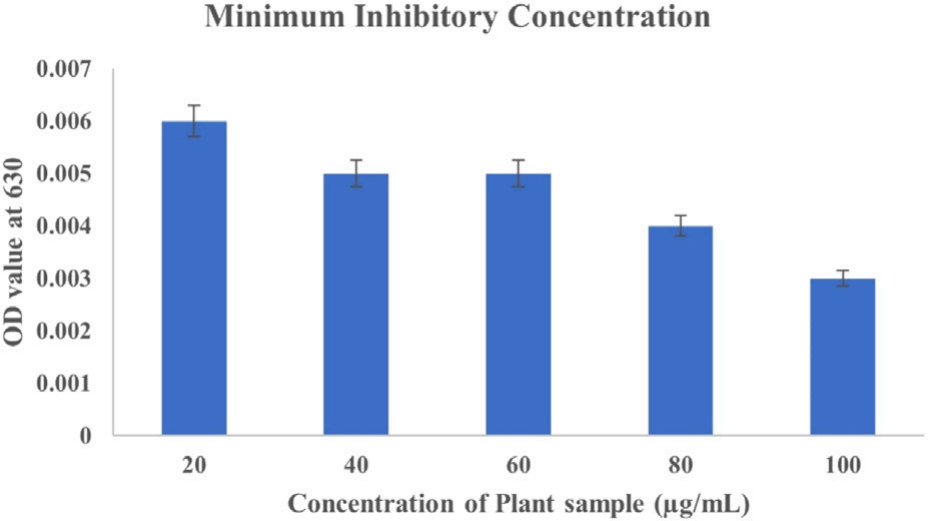
Graphical representation of the MIC (Broth Dilution Test)

Determining the Minimum Inhibitory Concentration (MIC) of *C. hirta* extract against *P. aeruginosa* provides valuable insight into its antibacterial potential. The MIC was established at 20 μg/mL, demonstrating significant inhibitory effects on bacterial growth, as evidenced by the minimal optical density (OD) 0.001. This finding underscores the efficacy of the bioactive compounds in the plant extract, which likely disrupt bacterial membranes and interfere with essential cellular processes, such as protein synthesis or enzymatic functions. Interestingly, while higher concentrations (40–100 μg/mL) showed increased OD values, particularly at 40–60 μg/mL (0.016), this does diminish the observed antibacterial potency at 80 and 100 μg/mL concentrations (Figure 1). The variation at higher concentrations could be attributed to multiple factors, including potential aggregation of phytochemicals, interaction with assay components, or saturation effects that do not necessarily reflect reduced efficacy. Such variations highlight the importance of considering biological and technical factors in interpreting MIC results. *Clidemia hirta* exhibited the CC_50_ value of 45.28 μg/mL against Vero cells (half of the Vero cells in the test were non-viable) and MIC ranged with the concentration of 20 μg/mL showed the MIC. The selective index was observed at 4.543, which indicates that the *C. hirta* shows moderate selectivity. It suggests that the compound has a reasonable balance of efficacy and safety, making it further use or development. The 70% ethanolic extract of *C. hirta* contains flavonoids, saponins, tannins, and triterpenoid compounds, according to Pratami et al. (2021). The aqueous extract also contains flavonoids, saponins, tannins, and steroids that can inhibit all concentrations of *S. typhi* and *S. aureus*. Still, the aqueous extracts inhibited bacterial growth only at 12.5% and 25% concentrations.

### 3.3 GC-MS

**FIGURE 2:**
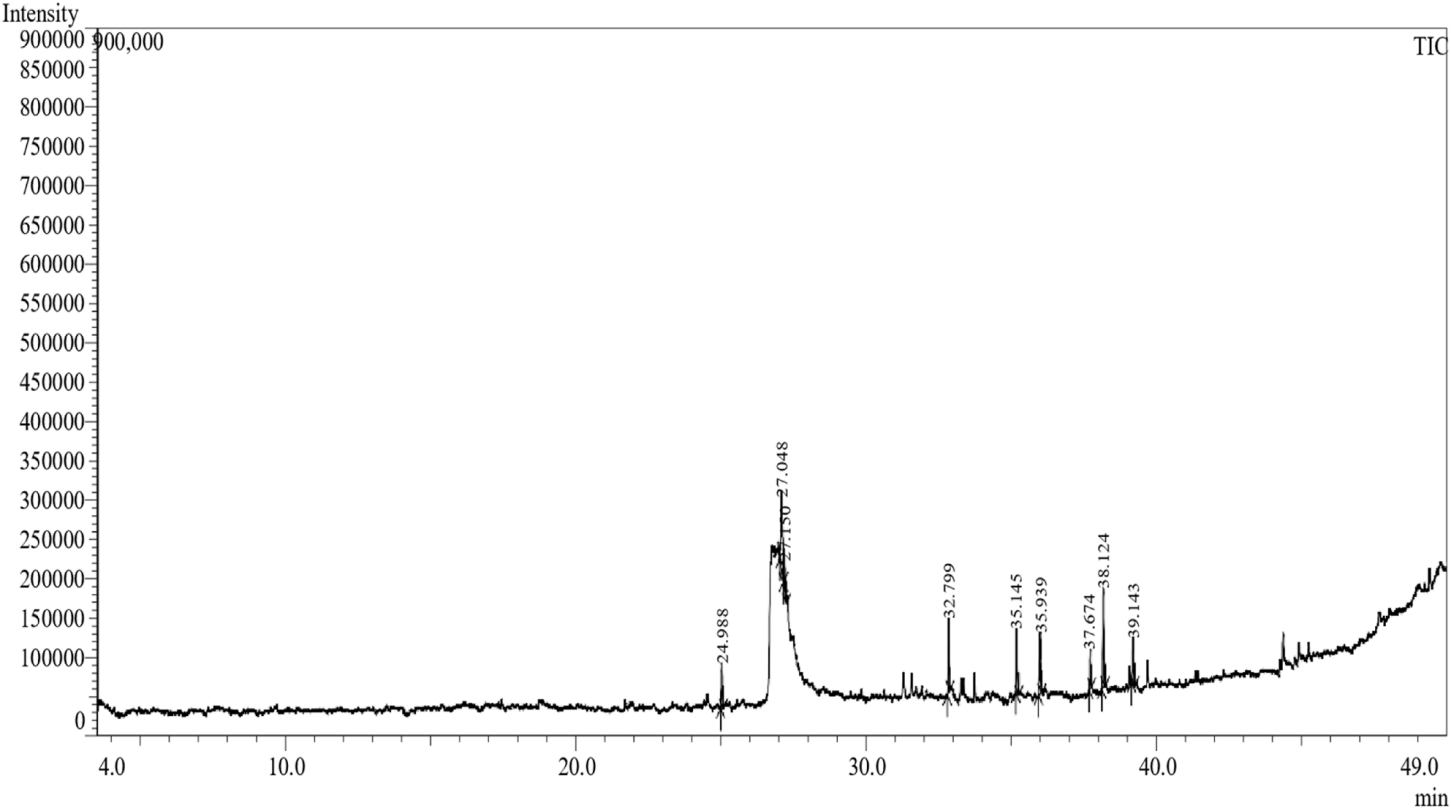
GCMS analysis of methanolic leaf extracts of *C. hirta*.

The methanol extract of the leaves contains nine high-peaked chemicals, including diethyl phthalate, 2,4-Di-tert-butylphenol, 1,2-Benzenedicarboxylic acid, DI, Neophytadiene, Dibutyl phthalate, Hexadecanoic acid, ethyl ester, behenic alcohol, 2-Hexadecen-1-OL, 3,7,11,15-Tetramethyl, and Ethyl Oleate (Figure 2; Table 1).

**TABLE 1:** Peaked compounds of the *C. hirta* from the GC-MS analysis.

Peak#	R. Time	Area	Area%	Name	Chemical structure
1	24.988	161,955	8.06	2, 4-Di-tert-butyl-phenol	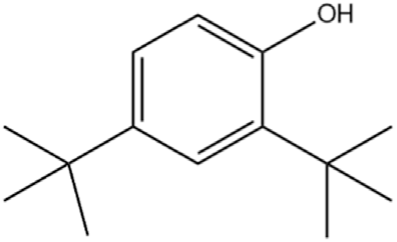
2	27.048	396,893	19.75	Diethyl Phthalate	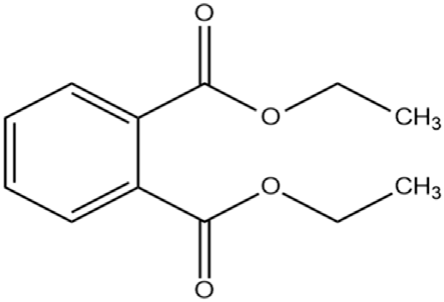
3	27.150	136,635	6.80	1, 2-Benzenedicarboxylic acid, DI	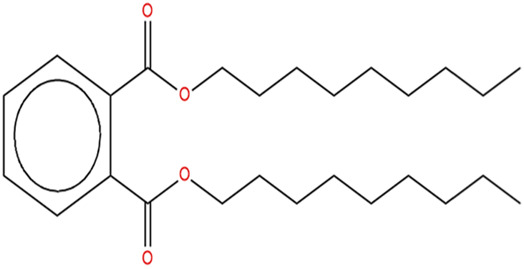
4	32.799	236,150	11.75	Neophytadiene	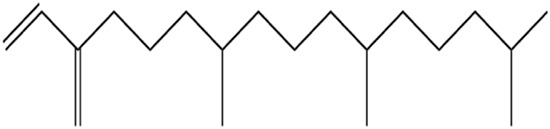
5	35.145	231,048	11.50	Dibutyl phthalate	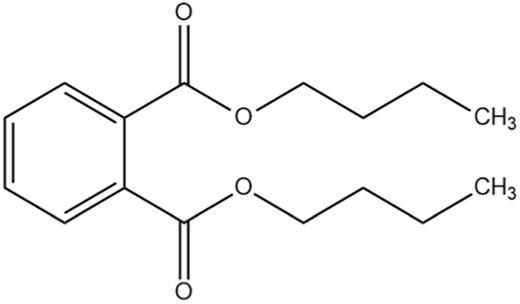
6	35.939	210,622	10.48	Hexadecanoic acid, ethyl ester	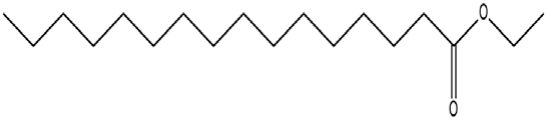
7	37.674	117,913	5.87	Behenic alcohol	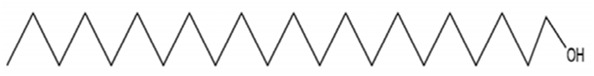
8	38.124	344,214	17.13	2-Hexadecen-1-OL, 3,7,11,15-Tetram	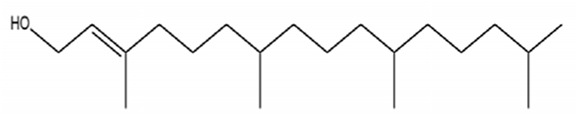
9	39.143	173,929	8.66	Ethyl Oleate	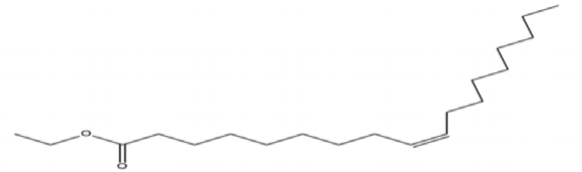

### 3.4 Molecular docking

The differences in binding affinity and ligand efficiency observed among the compounds can be attributed to the structural and physicochemical properties of the molecules, which influence their interactions with the target receptor. In this study, both 2, 4-Di-tert-butyl-phenol and Dibutyl phthalate demonstrate superior binding affinities, with values of −6.2 kcal/mol, signifying more robust interactions when compared to Diethyl phthalate, which exhibited a lower binding affinity of −5.7 kcal/mol. Given that the binding affinities and estimated Ki values for 2, 4-Di-tert-butyl-phenol and Dibutyl phthalate are equivalent, further evaluation utilizing additional parameters, such as ligand efficiency (−0.41 for 2, 4-Di-tert-butyl-phenol vs. −0.31 for Dibutyl phthalate), emphasizes the slightly higher efficiency of 2, 4-Di-tert-butyl-phenol. It exhibits a higher ligand efficiency (−0.41) than Dibutyl phthalate (−0.31) due to its smaller size and more favorable interactions with the receptor. The presence of bulky tert-butyl groups might provide additional van der Waals interactions or hydrophobic contacts, enhancing the binding strength without significantly increasing molecular complexity. Despite having the same binding affinity (−6.2 kcal/mol) as 2,4-Di-tert-butyl-phenol, its ligand efficiency is lower in Dibutyl phthalate. This suggests that its molecular structure may have fewer favorable interactions per unit of its molecular weight. Dibutyl phthalate likely forms effective van der Waals or hydrophobic interactions but lacks the same level of compact efficiency seen in 2,4-Di-tert-butyl-phenol. Diethyl phthalate has a lower binding affinity (−5.7 kcal/mol), likely due to its smaller alkyl groups (ethyl groups) compared to Dibutyl phthalate’s bulkier butyl groups (Table 2). The reduced bulk limits its hydrophobic surface area and, consequently, its interaction with hydrophobic regions of the receptor, leading to weaker binding. Overall, the enhanced performance of 2, 4-Di-tert-butyl-phenol arises from a combination of optimized hydrophobicity, molecular size, and shape, which contribute to its superior binding affinity and ligand efficiency. These results highlight the importance of structural features in modulating receptor-ligand interactions.

**TABLE 2:** Docking Score of the ligands against the target protein (7KIS).

Poses	Compound	Binding affinity (Kcal/Mol)	Estimated ki (µM)	Ligand efficiency
1	2,4-Di-tert-butyl-phenol	−6.2	28.53	−0.41
2		−6.1	33.78	−0.41
1	Dibutyl phthalate	−6.2	28.53	−0.31
2		−6.2	28.53	−0.31
1	Diethyl phthalate	−5.7	66.36	−0.36
2		−5.7	66.36	−0.36

High-affinity values show high interaction between the target protein and ligand.

### 3.5 *In silico* dynamics and simulation

**FIGURE 3:**
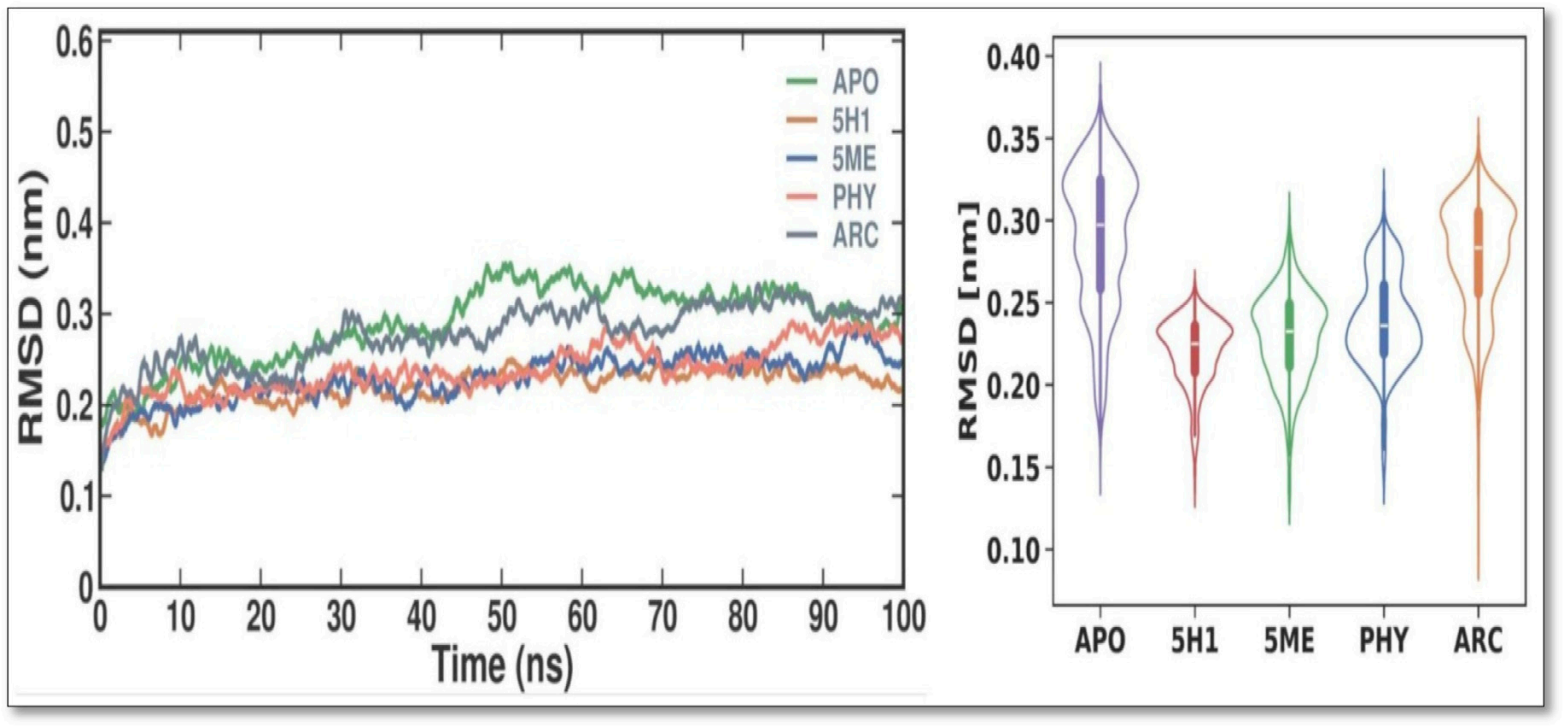
Conformational dynamics of 7KIS-APO, 7KIS-5H1, 7KIS-5ME, 7KIS-PHY, and 7KIS-ARC complex. Time evolution of the RMSD values (Left); Violin Plot of RMSD values (Right).

The RMSD values were examined throughout time (as illustrated in Figure 3) to examine the stability of the PBP2 and understand the systems’ behavior. The findings show that both systems were evenly dispersed throughout the simulation and reached equilibrium in 10 ns. Additionally, the evaluation of the RMSD values showed that the docked complex remained stable throughout the simulation, with the 7KIS-APO, 7KIS-5H1, 7KIS-5ME, 7KIS-PHY, and 7KIS-ARC complexes maintaining stability for up to 100 ns? Furthermore, compared to its free form, the RMSD pattern of 7KIS-APO was reduced following the binding of 2 ME, BEN, and ARC. This result implies that the docked complex of 7KIS-APO, 7KIS-5H1, 7KIS-5ME, 7KIS-PHY, and 7KIS-ARC is a stable system that did not show notable oscillations throughout the simulation. 7KIS-APO, 7KIS-5H1, 7KIS-5ME, 7KIS-PHY, and 7KIS-ARC were found to have average RMSD values of 0.29 ± 0.04 nm, 0.22 ± 0.02 nm, 0.23 ± 0.03 nm, 0.24 ± 0.03 nm, and 0.28 ± 0.03 nm, respectively.

**FIGURE 4:**
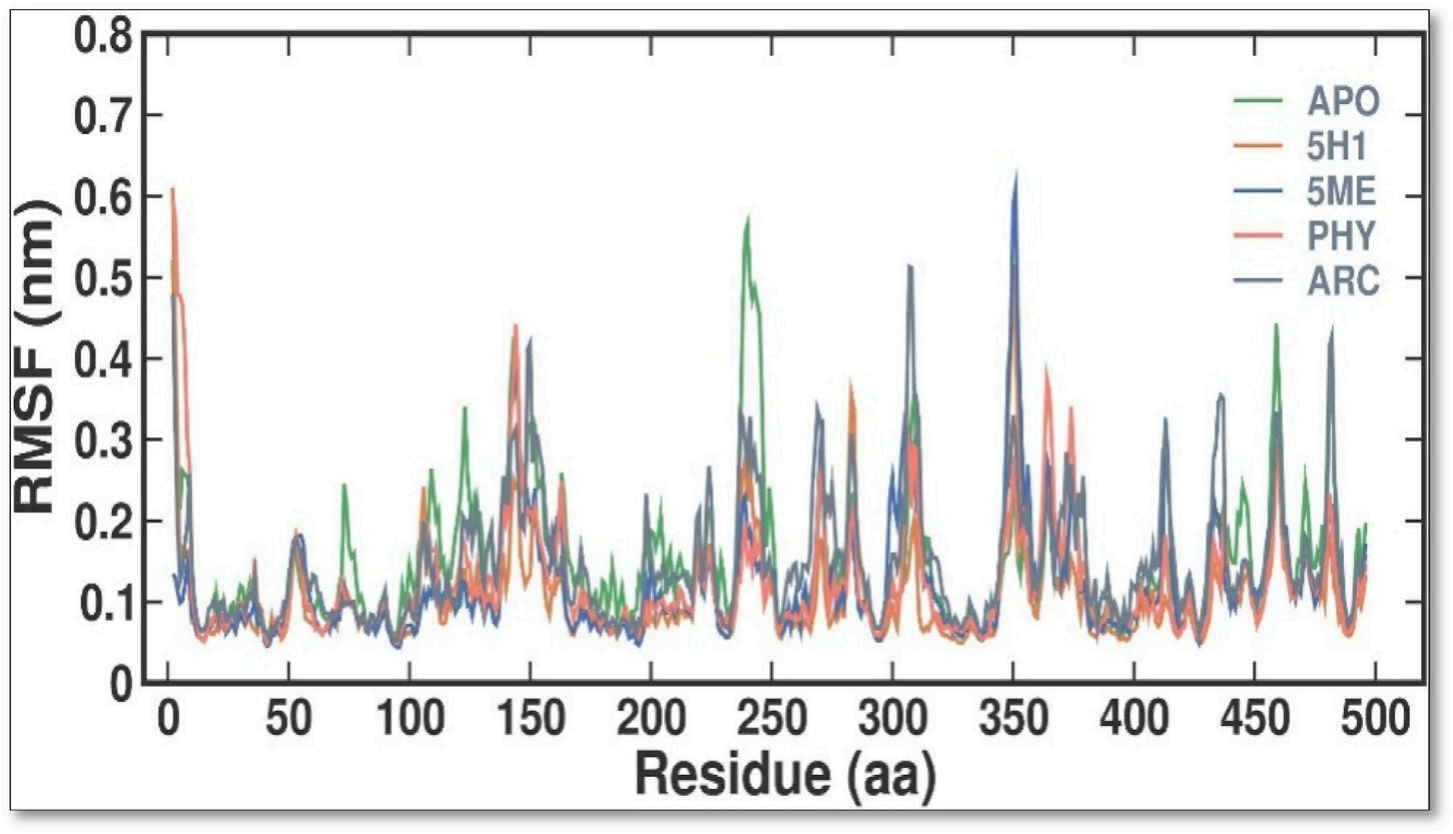
Conformational dynamics of 7KIS-APO, 7KIS-5H1, 7KIS-5ME, 7KIS-PHY, and 7KIS-ARC complex. Time evolution of the RMSF values.

During MD simulations, RMSF quantifies the variations of every residue and flexible area of a protein. It is possible to ascertain the effect of ligand binding on the protein by examining RMSF during simulations. Loosely organized loop sections generally have greater RMSF values than compact protein structures like sheets and helices. Every residue in 7KIS-APO had its RMSF values computed and plotted in this study, both before and after 7KIS-5H1, 7KIS-5ME, 7KIS-PHY, and 7KIS-ARC binding (Figure 4). According to the findings, the overall RMSF distribution was not substantially changed by the binding of APO, 5H1, 5 ME, PHY, and ARC. The 5H1, 5 ME, PHY, and ARC complexes likewise showed fewer oscillations than free APO. The results showed that the average RMSF values for APO, 7KIS-5H1, 7KIS-5ME, 7KIS-PHY, and 7KIS-ARC were, respectively, 0.15 ± 0.08 nm, 0.11 ± 0.06 nm, 0.12 ± 0.07 nm, 0.12 ± 0.07 nm, and 0.15 ± 0.08 nm.

**FIGURE 5:**
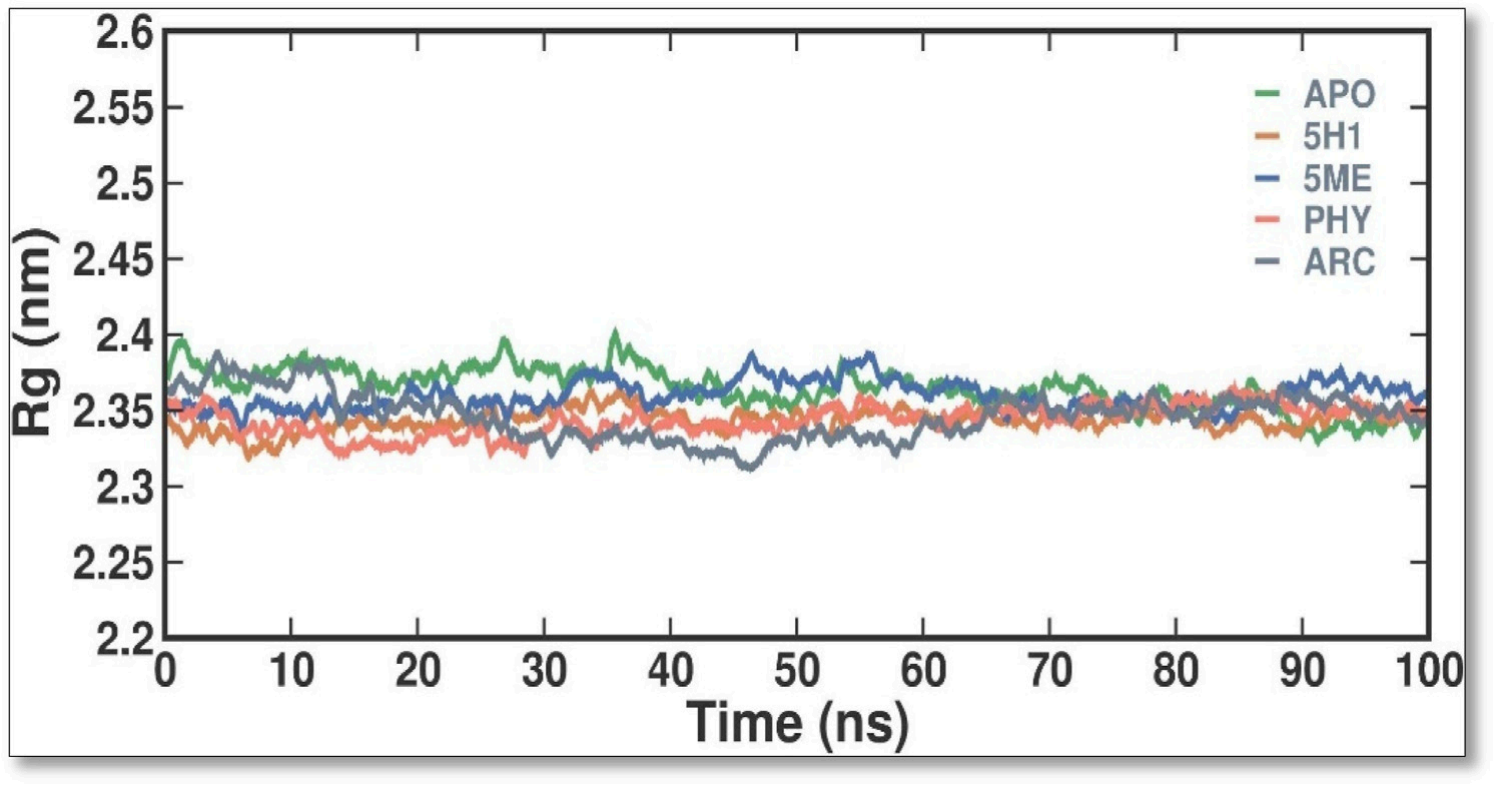
The conformational dynamics of the 7KIS-APO, 7KIS-5H1, 7KIS-5ME, 7KIS-PHY, and 7KIS-ARC complex revolve around the time evolution of the RG values.

The Rg values were calculated and plotted over time to evaluate the dynamic stability and compactness of APO and its APO, 5H1, 5 ME, PHY, and ARC complexes (Figure 5). Average Rg values were found to be 2.36 ± 0.02 nm, 2.34 ± 0.01 nm, 2.36 ± 0.01 nm, 2.34 ± 0.01 nm, and 2.35 ± 0.02 nm for APO, 7KIS-5H1, 7KIS-5ME, 7KIS-PHY, and 7KIS-ARC, respectively. Compared to the apo form of 7KIS, the complex system showed lower Rg values, suggesting it was more compact. The plot indicates that the docked complex of 2 ME, BEN, and ARC with 7KIS stayed stable during the simulation.

**FIGURE 6:**
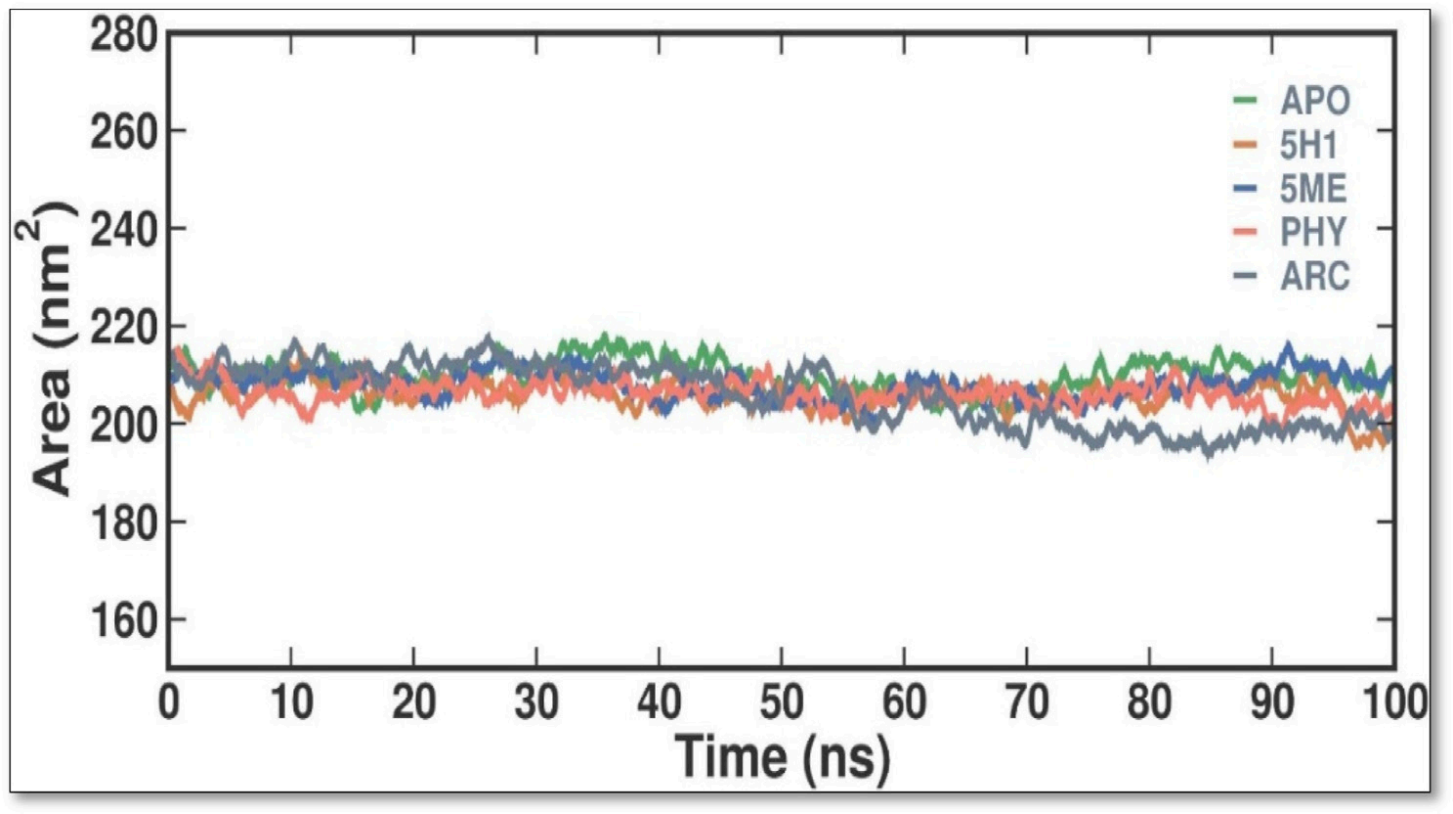
Conformational dynamics of 7KIS-APO, 7KIS-5H1, 7KIS-5ME, 7KIS-PHY, and 7KIS-ARC complex showed the time evolution of the SASA values.

SASA is a helpful metric for assessing a protein molecule’s accessibility in a solvent environment. The effect of 2 ME, BEN, and ARC binding on the solvent accessibility of 7KIS was evaluated in this investigation by computing and plotting the SASA values (Figure 6). When APO binds with 2 ME, BEN, and ARC, the plot shows a modest rise in its SASA values. Accordingly, some of the protein’s internal residues might be visible on the surface when ligands bind to it. Throughout the simulation, there are no notable variations in the SASA values, which indicate fair equilibration.

**FIGURE 7:**
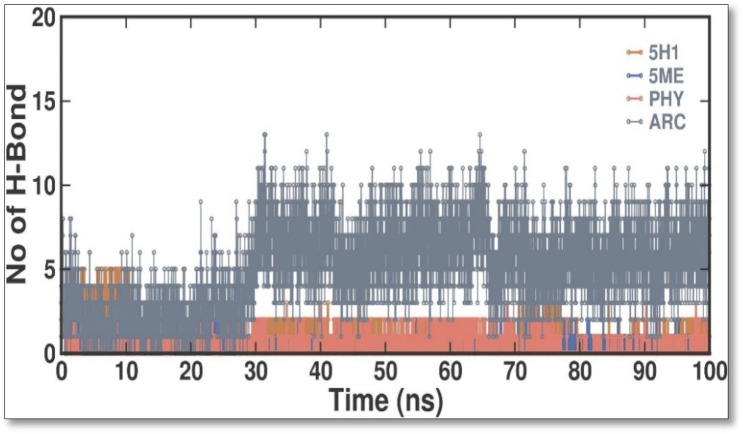
Intermolecular H bonds between 5H1, 5 ME, PHY, and ARC during the simulation. Principal component analysis 2D projection plot shows the conformation sampling of APO, 5H1, 5 ME, PHY, and ARC on PC1 and PC2.

When evaluating the stability of protein-ligand interactions, creating H bonds is essential. The time-dependent behavior of the H bonds between 2 ME, BEN, and ARC was examined in this work (Figure 7). The plot showed that up to 4 H bonds were established between APO, 5H1, 5 ME, PHY, and ARC, despite more significant variations. According to our study, at least 1–5 H bonds with 5H1, 1-13 with 5 ME, 1-5 with PHY, and 1-12 with ARC kept the docked complex stable throughout the simulation.

**FIGURE 8:**
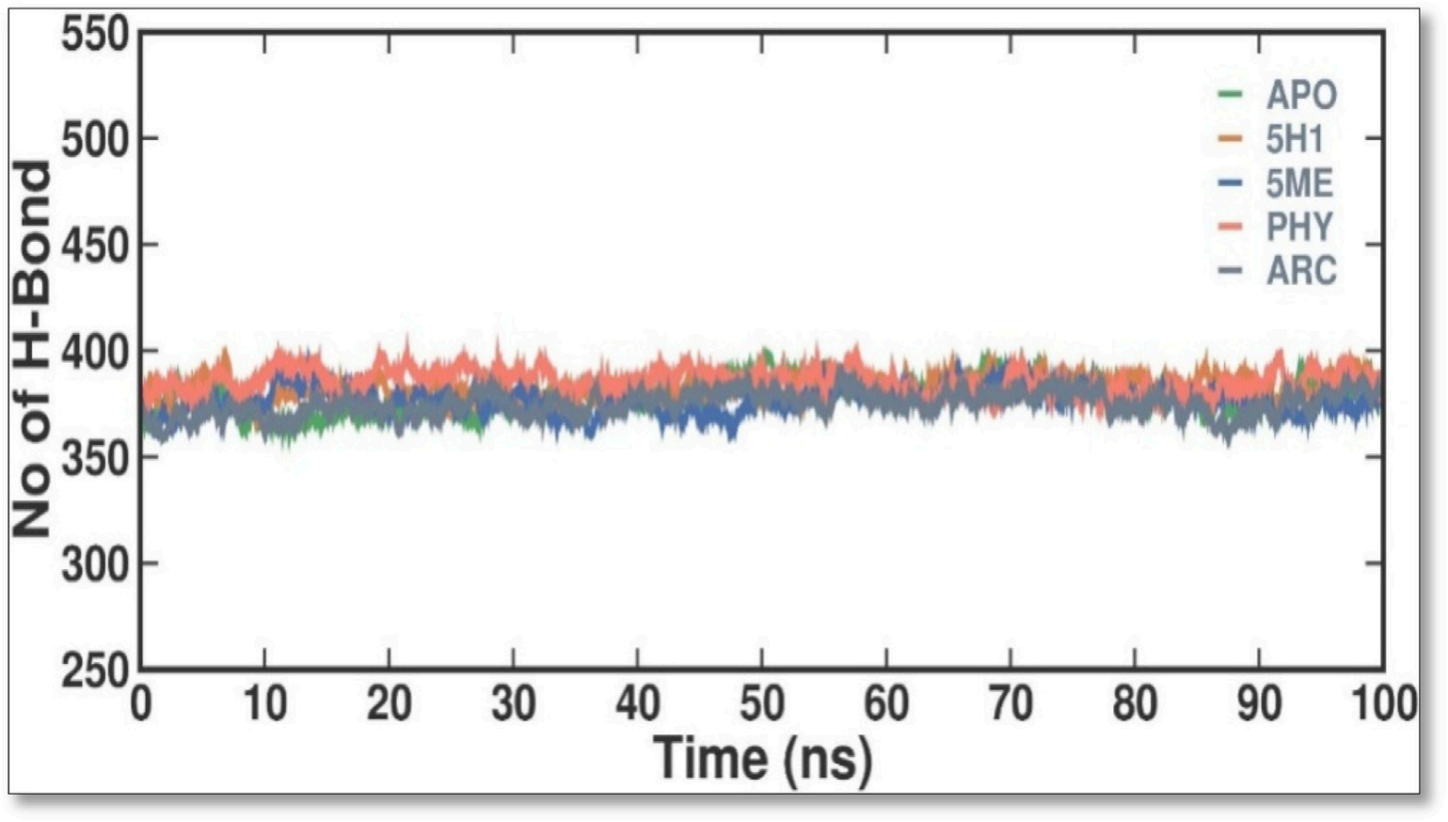
Intermolecular H bonds between APO, 5H1, 5 ME, PHY, and ARC in simulation. The principal component analysis 2D projection plot shows APO, 5H1, 5 ME, PHY, and ARC conformation sampling on PC1 and PC2.

The PCA was performed to investigate the collective motions in the APO, 5H1, 5 ME, PHY, and ARC complexes. The five eigenvectors (EVs) are essential for a protein molecule’s overall motion. To investigate the conformational dynamics of the APO and APO, 5H1, 5 ME, PHY, and ARC complexes during the simulation, we employed PCA. The PCA time evolution indicates stability by indicating a decrease in the overall flexibility of the APO, 5H1, 5 ME, PHY, and ARC complex on both EVs. The APO, 5H1, 5 ME, PHY, and ARC complexes filled nearly all conformational movements and overlapped, as the plot shows (Figure 8). Overall, the stability of the complex is supported by the lower number of movements seen in the 2 ME complex, which indicates that 2 ME had no discernible impact on the 7KIS structure and dynamics.

### 3.6 MM - PBSA

APO, 7KIS-5H1, 7KIS-5ME, 7KIS-PHY, and 7KIS-ARC binding affinities were assessed by analyzing the relative binding strength within the protein of summery energy. Table 3 contrasts APO, 7KIS-5H1, 7KIS-5ME, 7KIS-PHY, and 7KIS-ARC binding strengths with inhibitors calculated using the MM-PBSA method. We compute contributions to the interaction energy at the residue level across a steady simulation trajectory.

**TABLE 3:** Comparison of the binding strength of APO, 7KIS-5H1, 7KIS-5ME, 7KIS-PHY, and 7KIS-ARC concerning inhibitors computed via the MM-PBSA method.

System	Van der waal (kJ/mol)	Electrostatic (kJ/mol)	Polar solvation (kJ/mol)	Binding (kJ/mol)
7KIS-5H1	−106.846 ± 16.242	−20.754 ± 8.133	69.416 ± 15.761	−70.007 ± 12.971
7KIS-5ME	−152.287 ± 17.947	−8.526 ± 3.645	73.079 ± 9.207	−103.396 ± 21.367
7KIS-PHY	−136.377 ± 12.477	−5.848 ± 7.058	58.695 ± 15.057	−101.191 ± 15.845
7KIS-ARC	−265.600 ± 16.280	−107.006 ± 20.892	325.010 ± 43.566	−77.507 ± 13.443

According to the findings, 7KIS-5H1 has a binding energy of −70.007 ± 12.971 kJ/mol, a polar solvation energy of 69.416 ± 15.761 kJ/mol, an electrostatic energy of −20.754 ± 8.133 kJ/mol, and a van der Waals energy of −106.846 ± 16.242 kJ/mol. The energy of 7KIS-5ME is −152.287 ± 17.947 kJ/mol for the van der Waals, −8.526 ± 3.645 kJ/mol for the electrostatic, 73.079 ± 9.207 kJ/mol for the polar solvation, and −103.396 ± 21.367 kJ/mol for the binding. An electrostatic energy of −5.848 ± 7.058 kJ/mol, a polar solvation energy of 58.695 ± 15.057 kJ/mol, a binding energy of −101.191 ± 15.845 kJ/mol, and a van der Waals energy of −136.377 ± 12.477 kJ/mol are all present in 7KIS-PHY. The energy values of 7KIS-ARC are as follows: polar solvation energy: 325.010 ± 43.566 kJ/mol; binding energy: 77.507 ± 13.443 kJ/mol; van der Waals energy: 265.600 ± 16.280 kJ/mol; electrostatic energy: 107.006 ± 20.892 kJ/mol. More analysis would be required to make more thorough inferences regarding the data.

## 4 Discussion

The antimicrobial compounds derived from *C. hirta* exhibit a multifaceted mechanism of action, initiating their effect at the bacterial outer membrane by targeting lipopolysaccharides and phospholipids. This interaction compromises membrane integrity, leading to pore formation that facilitates the intracellular penetration of antibiotics. The disruption of the outer membrane, coupled with enhanced permeability, amplifies the efficacy of antibiotics such as β-lactams and glycopeptides, which inhibit bacterial cell wall synthesis. This synergistic interaction weakens the bacterial defence system, allowing antimicrobial agents to infiltrate the cytoplasm more effectively. Within the cytoplasm, these agents bind to the 16 S rRNA of the 30 S ribosomal subunit, predominantly near the A-site, through H bonding (Halawa et al., 2024). This binding perturbs ribosomal fidelity, causing translational errors, premature termination of mRNA decoding, or complete inhibition of protein synthesis. Disruption of critical protein synthesis pathways leads to cellular dysfunction and eventual bacterial death. Moreover, the membrane-disruptive properties of *C. hirta* antimicrobials likely compromise the function of efflux pumps, further enhancing intracellular retention of both the antimicrobial compounds and co-administered antibiotics. Combining cell wall synthesis inhibition with ribosomal interference, this dual—action strategy renders these compounds exceptionally potent against resistant bacterial strains. The synergistic effect of these mechanisms underscores the therapeutic potential of *C. hirta* extracts, offering a promising avenue for augmenting antibiotic efficacy, particularly in the context of combating multidrug-resistant pathogens (Kapoor et al., 2017).

The pronounced antibacterial activity observed at a low MIC suggests that *C. hirta* possesses potent bioactive compounds synergize within the extract and may disrupt the bacterial cell wall or penetrate the cytoplasm to inhibit ribosomal functions, as hypothesized in other studies of plant-based antimicrobials. The results align with previous findings on the antibacterial properties of medicinal plants, reinforcing the potential of *C. hirta* as a source of novel antimicrobial agents. Against every bacterial strain examined, *C. hirta* leaf extracts demonstrated an antibacterial activity. The presence of highly active ingredients or compounds in the extract is indicated by the very low extract concentration (0.7–2.2 mg mL^−1^) needed for the complete suppression of bacterial growth (MBC), regardless of the strain (Lopez et al., 2016). The compounds with high peaks were reported on GCMS analysis and could provide deeper insights into the mechanisms underlying their antibacterial efficacy. Hexadecanoic acid, ethyl ester, behenic alcohol, 2-hexadecen-1-OL, 3, 7, 11, 15-tetram, ethyl phthalate, 1, 2-benzenedicarboxylic acid, DI, Neophytadiene, and ethyl phthalate were all noted.

2, 4-di-tert-butylphenol, also known as 2, 4-bis (1,1-dimethyl ethyl)-phenol (DTBP or 2, 4-DTBP), is a standard natural product that is highly toxic to nearly all testing organisms, including the producing species. It is well known to have various biological effects due to its antifungal, antibiotic, antibacterial, insecticidal, and antioxidant properties. It is also a common secondary metabolite that is produced by different groups of organisms (Dharni et al., 2014; Belghit et al., 2016; Zhao et al., 2020; Mishra et al., 2020; Lee et al., 2024). The compounds have a universal role in inhibiting the pathogenic bacterial communities, according to a report on DTBP extraction from the thermophilic bacteria *Bacillus licheniformis* (Aissaoui et al., 2018). Additionally, Song et al. (2018) reported that 2,4 di tert butylphenol (DTBP) induces senescence and mitotic catastrophe in human gastric adenocarcinoma AGS cells, which may be due to inhibition of HDAC6 (Histone deacetylases) enzyme activity. DTBP possesses the inhibitory enzymatic activity reported in bamboo shoots on α-glucosidase and α-amylase (Sansenya et al., 2023). Compound Diethyl phthalate has antimicrobial, acetylcholinesterase, and neurotoxic activity (Velanganni et al., 2011), covering 19.75% in the current study. The compound Di-butyl phthalate, isolated from *Begonia malabarica*, has been found to have vigorous antibacterial activity (Shobi and Viswanathan, 2018). At a concentration of 100 mg mL^−1^, it demonstrated 9 mm ZoI against *Staphylococcus epidermidis*, *Streptococcus pneumoniae*, *Escherichia coli*, *Micrococcus luteus*, *Klebsiella pneumoniae*, *Shigella flexneri*, *Vibrio cholerae*, and *P. aeruginosa*. Often referred to as PAEs, phthalate esters, or simply phthalates, phthalic acid esters (dialkyl or alkyl aryl esters of 1, 2-benzenedicarboxylic acid) are a class of significant phthalic acid derivatives that are produced by Fischer esterification from phthalic anhydride and particular alcohols (Gomez-Hens and Aguilar-Caballos, 2003). Covers of 1, 2-Benzenedicarboxylic acid When tested at 1,000 μg/mL against *S. typhi*, *E. coli*, *Streptococcus feacalis*, *Staphylococcus aureus*, *Candida krusei*, and *Shigella dysenteriae*, respectively, 6.80% of the samples with diameters of 20, 16, 28, 24, 29, and 22 mm achieved the ZoI (Shoge et al., 2016). The antibacterial and antioxidant properties of volatile oils extracted from the fresh blooms of three buckwheat plants—*Fagopyrum esculentum*, *Fagopyrum tataricum*, and *Fagopyrum cymosum*—were investigated by Zhao et al. (2018). They found that 13.19% of the volatile oils included molecule 1, 2-Benzenedicarboxylic acid. *Trichoderma indicum* aerial parts hexane extract showed vigorous anti-inflammatory activity in RAW264.7 cells; specifically, Fraction E (FE) demonstrated direct target activity against COX-2, NO, IL-1β, TNF-α, and iNOS. With good inhibition of TLR-4, TACE, and NIK, 1, 2-benzene dicarboxylic acid di iso octyl ester was found to be a significant potential bioactive molecule (Hamsalakshmi et al., 2021). Phytoconstituents isolated from *Onosma bracteata*, specifically 1, 2-Benzenedicarboxylic acid, bis (2-methyl propyl) ester (BDCe fraction), have been shown by Kumar et al. (2022) to have enormous antiproliferative potential. These phytoconstituents target inflammatory, antiapoptotic, and proliferation markers and induce apoptosis in osteosarcoma cells. The marine-derived actinomycete *Streptomyces* sp. is the only source of the pure chemical 1, 2-benzene dicarboxylic acid, mono 2-ethyl hexyl ester (DMEHE), which exhibits cytotoxic action against HepG2 and MCF-7 cancer cell lines (Krishnan et al., 2014). The methanolic extracts of *Crataeva nurvala* and *Blumea lacera*, plants with sedative, depressive, and anxiolytic properties, contain a diterpene lactone called neophytadiene (NPT) (Gonzalez-Rivera et al., 2023). The same was discovered by Swamy et al. (2017) in certain plants, such as *Plectranthus amboinicus*, which is thought to have anti-inflammatory and antioxidant properties to support further According to Muhizi et al. (2005), leaf extracts from *Leonotis leonorus* demonstrated up to 50% protection against PTZ-induced seizures at a high dose of 400 mg/kg (i.p.); nevertheless, the mechanism behind the anticonvulsant action remained unclear. Hexadecanoic acid, ethyl ester, is a saturated fatty acid with a variety of properties, including antibacterial, anticancer, antioxidant, and antihemolytic properties (Agoramoorthy et al., 2007; Wei et al., 2011; Musa et al., 2015). According to Shaaban et al. (2021), Hexadecanoic acid methyl ester is multidrug-resistant against the pathogenic microorganisms *K. pneumoniae* DF30, *P. aeruginosa* D31, *K. pneumoniae* B40, and *S. aureus* W35. *Imperata cylindrica* contains volatile chemicals, such as hexadecanoic acid methyl ester (35.8%), which has antibacterial properties against tested bacteria, including *K. pneumoniae*, *B. subtilis*, and *P. aeruginosa* (Lalthanpuii and Lalchhandama, 2019). Davoodbasha et al. (2018) also found that *Scenedesmus intermedius* contained hexadecanoic acid methyl ester (24.08%), which showed a strong inhibitory impact against Gram-positive and Gram-negative bacteria. Anneken et al. (2006) indicate that hexadecanoic acid methyl ester is a fatty acid ester known to inhibit the growth of harmful bacteria. The antibacterial activity of fatty acids, including hexadecanoic acid methyl ester, is influenced by the structure, form, and function of their carbon chains, as well as the presence, quantity, location, and orientation of double bonds. Various species utilize the antibacterial properties of fatty acid methyl esters to protect themselves from pathogens. The main target of these compounds is the bacterial cell membrane, where they hinder enzyme activity, disrupt cellular energy production, and ultimately induce bacterial cell lysis. Due to its safety and efficacy, Suresh et al. (2014) highlight fatty acid methyl ester as a promising antibacterial agent. 2-hexadecen-1-ol, 3,7,11,15-tetramethyl also known as Phytol, naturally occurring compound present in chlorophyll and commonly used as a precursor for the synthesis of vitamins E and K. It has been investigated for its role in regulating serum urate levels and exhibits various biological activities, including antioxidant effects, anti-inflammatory properties, and the potential to inhibit cholinesterase (Elufioye et al., 2017). EL-fayoumy et al. (2021) found that the pure components of *Chlorella vulgaris* ((2E,7R, 11R)-3,7,11,15-Tetramethyl-2-hexadecenol) display significant antioxidant and anticancer activities when compared to synthetic and natural standards. Furthermore, the methanolic extract of the stem and root bark of *Kirganelia reticulata* contains similar compounds that possess anticancer properties (Reddy et al., 2017). Mohamed et al. (2015) also demonstrated that the crude extract of *Nitraria retusa* exhibits comparable chemical, anticancer, and antioxidant properties. Phytochemical research has identified the presence of flavonoids, saponins, triterpenoids, and steroid compounds with antibacterial properties in *C. hirta* Specific secondary metabolites can regulate the growth and development of various species; for instance, the metabolism of pyruvate and sulfur in plants significantly influences the production of arillus, particularly in *Salacca sumatrana* (Fendiyanto et al., 2020; Fendiyanto et al., 2021). Additionally, genetic factors play a crucial role in the metabolic processes of some organisms; specifically, SnpB11 and OsGERLP are involved in metabolic regulation that helps mitigate aluminium stress (Fendiyanto et al., 2019). By utilizing biological markers, researchers can focus their investigation on *C. hirta*’s secondary metabolites to understand the mechanisms underlying the interaction between these extracts and microbial growth. Some biological markers may be categorized into several types, including metabolite markers, physiological markers (Andriyanto and Fendiyanto, 2019; Miftahudin et al., 2021), genetic markers (Satrio et al., 2019; Pratami et al., 2020), or morphological markers (Pratami et al., 2019). It needs to be also considered that the phytocomponent composition of ethanolic extracts from different plant genotypes vary, and their bioactivity profiles vary (Cavar Zeljkovic et al., 2022; Schadich et al., 2025). Further studies are needed to assess whether the antibacterial property vary among different chemovars and genotypes. Certain active compounds known to act as antibacterial agents also exhibit specificity. For example, alkaloids possess antimicrobial properties due to their ability to intercalate DNA. Phytochemical tests have shown that the phenolic components of the samples include tannins, which can disrupt protein membranes and deactivate enzyme proteins, thereby exhibiting antibacterial qualities (Pelezar and Chan, 1988; Scalbert, 1991; Puupponen-Pimia et al., 2001; Xu et al., 2015). Meanwhile, saponins are surface-active substances that create soap foam when dissolved in water and may compromise the permeability of bacterial cell membranes (Murphy, 1999).

In our study, the identified compounds have a paramount efficacy against many multi-resistant pathogenic bacteria, exclusively *P. aeruginosa*. Moreover, the extract’s effectiveness against *P. aeruginosa*, a pathogen with notable resistance to conventional antibiotics, suggests a potential role in addressing the rising global challenge of antimicrobial resistance. Future research should explore the synergistic effects of *C. hirta* extract with existing antibiotics and its *in vivo* efficacy with safety profile. These studies could pave the way for its incorporation into alternative or adjunctive antimicrobial therapy. The present findings strongly advocate for developing plant-derived antimicrobial strategies as sustainable solutions to combat resistant bacterial pathogens. To further the study, a combination of mathematical techniques—such as quantitative structure-activity relationship (QSAR) analysis, homology modelling, docking simulations, ADMET assessments, and molecular dynamics simulations—was employed to identify strong and non-toxic aminoguanidine derivatives that have previously been evaluated as inhibitors of *P. aeruginosa*. Utilizing bioavailability radar approaches, compounds 15 and 18 emerged as the most promising candidates for RNA polymerase-binding transcription factor and as inhibitors of the SARS-CoV-2 virus primary protease, respectively, based on docking simulations that indicated maximal binding affinity. While general testing (both *in vitro* and *in vivo*) is warranted to validate our theoretical findings, molecular dynamics simulations verified the stability of their binding interactions with the modelled proteins throughout the simulation duration, as indicated by RMSD, RMSF, and SASA analyses. These investigations could prove valuable in searching for new treatments targeting the main protease of the SARS-CoV-2 virus and *P. aeruginosa* (Edche et al., 2022). The molecular docking and dynamics processes for *C. hirta* against *P. aeruginosa* yielded favourable binding energies and excellent docking scores. The simulations demonstrated stability, reduced fluctuation, compactness, and the influence of the compounds on the protein through analysis of RMSD, RMSF, RG, SASA, and H bond parameters. In a further study, a combination of mathematical techniques—such as quantitative structure-activity relationship (QSAR) analysis, homology modelling, docking simulations, ADMET assessments, and molecular dynamics simulations—was employed to identify strong and non-toxic aminoguanidine derivatives that have previously been evaluated as inhibitors of *P. aeruginosa*. Utilizing bioavailability radar approaches, compounds 15 and 18 emerged as the most promising candidates for RNA polymerase-binding transcription factor and as inhibitors of the SARS-CoV-2 virus primary protease, respectively, based on docking simulations that indicated maximal binding affinity. While general testing (both *in vitro* and *in vivo*) is warranted to validate our theoretical findings, molecular dynamics simulations verified the stability of their binding interactions with the modeled proteins throughout the simulation duration, as indicated by RMSD, RMSF, and SASA analyses. These investigations could prove valuable in searching for new treatments targeting the main protease of the SARS-CoV-2 virus and *P. aeruginosa* (Edche et al., 2022). The molecular docking and dynamics processes for *C. hirta* against *P. aeruginosa* yielded favorable binding energies and excellent docking scores. The simulations demonstrated stability, reduced fluctuation, compactness, and the influence of the compounds on the protein through analysis of RMSD, RMSF, RG, SASA, and hydrogen bond parameters.

## 5 Conclusion

In the findings, *in vitro* assays confirmed the phytochemicals in the leaf extract exhibited potent inhibitory effects on bacterial growth, as evidenced by the clear inhibition zones. In MIC, the inhibitions were observed at 20 μg/mL and as gradual increase the bacterial concentration were diminish at 80 and 100 μg/mL concentrations with the SI of 4.543, suggesting the compound has moderately selective. In the study of molecular docking and dynamics, compounds 2,4-Di-tert-butyl-phenol and Dibutyl phthalate showed the remarkable binding affinity (−6.2 kcal/mol) towards the receptor of PBP2a in *P. aeruginosa*. The 2,4-Di-tert-butyl-phenol possess good hydrophobicity, molecular size, and shape, which provide higher affinity towards the receptor with remarkable ligand efficiency. From the RMSD result, the 7KIS-APO, 7KIS-5H1, 7KIS-5ME, 7KIS-PHY, and 7KIS-ARC systems were found to be stable throughout the simulation. The RMSF, Rg, SASA and H bonding results suggest that there were no structural changes in the protein with both the systems being structurally stable. Considering all these, the compounds 2,4-Di-tert-butyl-phenol and Dibutyl phthalate can be the better candidates taken for further *in vivo* studies. Future investigations could utilize advanced methods employing omics technologies—including proteomics and metabolomics—could offer deeper insights into the molecular mechanisms underlying the antibacterial action of these phytochemicals. Assessing the extract’s efficacy *in vivo* through animal model studies will be essential for validating its therapeutic potential and safety. These pathways will bridge the gap between laboratory results and clinical applications, paving the way for developing potent, plant-based antibacterial therapies.

## Data Availability

The raw data supporting the conclusions of this article will be made available by the authors, without undue reservation.
